# Supplementary material for: Micromix: web infrastructure for visualizing and remixing microbial ‘omics data
Source: Gigascience. 2025 Feb 3;14:giae120. doi: 10.1093/gigascience/giae120 (PMC11788673; doi:10.1093/gigascience/giae120)
Supplement: giae120_GIGA-D-24-00283_Original_Submission [file giae120_giga-d-24-00283_original_submission.pdf]

GigaScience

Micromix: web infrastructure for visualizing and remixing microbial ‘omics data

--Manuscript Draft--

**Manuscript Number:**	GIGA-D-24-00283
**Full Title:**	Micromix: web infrastructure for visualizing and remixing microbial ‘omics data
**Article Type:**	Research
**Funding Information:**	Natural Science and Engineering Research Council (CA) (RGPIN-2024-04305) — Prof. Lars Barquist<br>Bayerisches Staatsministerium für Wissenschaft, Forschung und Kunst (bayresq.net) — Prof. Lars Barquist
**Abstract:**	Micromix is a flexible web platform for sharing and integrating microbial ‘omics data, including RNA-seq and transposon-insertion sequencing. Currently, the lack of solutions for making data web-accessible results in ‘omics data being fragmented across supplementary spreadsheets or languishing as raw read data in public repositories. Micromix solves this problem, and can be easily deployed on a standard web server or using cloud services. It is organism-agnostic, accommodates data and annotations from various sources, and allows filtering based on KEGG pathways, GO terms, and curated gene sets. Visualizations are provided through a plug-in system that integrates existing visualization services and allows rapid development of new services, with available plug-ins currently supporting interactive heatmap and clustering functions. Users can upload their own data in a variety of formats to perform integrative analyses in the context of existing datasets. To support collaborative research, Micromix allows sharing of interactive sessions that maintain defined filtering and/or visualization options. We demonstrate the utility of Micromix with case studies focusing on the SPI-2 pathogenicity island in Salmonella enterica and polysaccharide utilization loci in Bacteroides thetaiotaomicron, showcasing the platform's capabilities for integrating, filtering and visualizing diverse functional genomic datasets. Micromix is available at http://micromix.systems.
**Corresponding Author:**	Lars Barquist<br>Helmholtz Institute for RNA-based Infection Research: Helmholtz-Institut fur RNA-basierte Infektionsforschung<br>Würzburg, GERMANY
**Corresponding Author Secondary Information:**	
**Corresponding Author's Institution:**	Helmholtz Institute for RNA-based Infection Research: Helmholtz-Institut fur RNA-basierte Infektionsforschung
**Corresponding Author's Secondary Institution:**	
**First Author:**	Regan J. Hayward
**First Author Secondary Information:**	
**Order of Authors:**	Regan J. Hayward
	Titus Ebbecke
	Hanna Fricke
	Vo Quang Nguyen
	Lars Barquist
**Order of Authors Secondary Information:**	
**Additional Information:**	
**Question**	**Response**

Powered by Editorial Manager® and ProduXion Manager® from Aries Systems Corporation

Are you submitting this manuscript to a special series or article collection?	No
**Experimental design and statistics**<br><br>Full details of the experimental design and statistical methods used should be given in the Methods section, as detailed in our Minimum Standards Reporting Checklist. Information essential to interpreting the data presented should be made available in the figure legends.<br><br>Have you included all the information requested in your manuscript?	Yes
**Resources**<br><br>A description of all resources used, including antibodies, cell lines, animals and software tools, with enough information to allow them to be uniquely identified, should be included in the Methods section. Authors are strongly encouraged to cite Research Resource Identifiers (RRIDs) for antibodies, model organisms and tools, where possible.<br><br>Have you included the information requested as detailed in our Minimum Standards Reporting Checklist?	Yes
**Availability of data and materials**<br><br>All datasets and code on which the conclusions of the paper rely must be either included in your submission or deposited in publicly available repositories (where available and ethically appropriate), referencing such data using a unique identifier in the references and in the "Availability of Data and Materials" section of your manuscript.<br><br>Have you have met the above requirement as detailed in our Minimum	Yes

Standards Reporting Checklist?	

Powered by Editorial Manager® and ProduXion Manager® from Aries Systems Corporation

Manuscript

Click here to access/download;Manuscript;Micromix_submission.docx

Micromix: web infrastructure for visualizing and remixing microbial ‘omics data

Regan J. Hayward[1†], Titus Ebbecke[1†], Hanna Fricke[1], Vo Quang Nguyen[1], Lars Barquist[1,2,3*]

[1] Helmholtz Institute for RNA-based Infection Research (HIRI), Helmholtz Centre for Infection Research (HZI), Würzburg, Germany.

[2] Faculty of Medicine, University of Würzburg, Würzburg, Germany.

[3] Department of Biology, University of Toronto, Mississauga, Canada

† Authors contributed equally to this work

* To whom correspondence should be addressed: lars.barquist@helmholtz-hiri.de

Graphical abstract

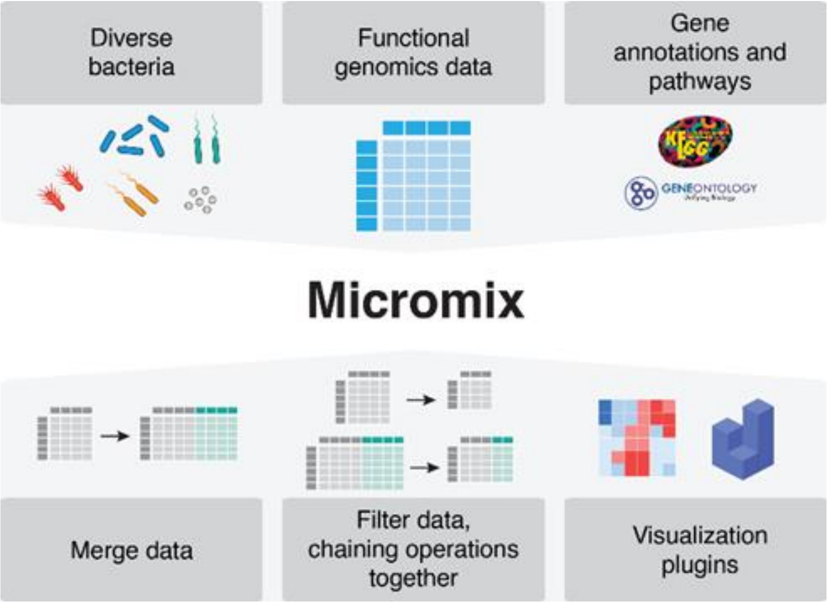

Abstract

Micromix is a flexible web platform for sharing and integrating microbial ‘omics data, including RNA-seq and transposon-insertion sequencing. Currently, the lack of solutions for making data web-accessible results in ‘omics data being fragmented across supplementary spreadsheets or languishing as raw read data in public repositories. Micromix solves this problem, and can be easily deployed on a standard web server or using cloud services. It is organism-agnostic, accommodates data and annotations from various sources, and allows filtering based on KEGG pathways, GO terms, and curated gene sets. Visualizations are provided through a plug-in system that integrates existing visualization services and allows rapid development of new services, with available plug-ins currently supporting interactive heatmap and clustering functions. Users can upload their own data in a variety of formats to perform integrative analyses in the context of existing datasets. To support collaborative research, Micromix allows sharing of interactive sessions that maintain defined filtering and/or visualization options. We demonstrate the utility of Micromix with case studies focusing on the SPI-2 pathogenicity island in *Salmonella enterica* and polysaccharide utilization loci in *Bacteroides thetaiotaomicron*, showcasing the platform's capabilities for integrating, filtering and visualizing diverse functional genomic datasets. Micromix is available at http://micromix.systems.

Introduction

Functional genomics technologies have generated vast amounts of data, enabling exploration of a wide range of cellular processes and interactions at a genome-scale (1, 2). For just a few examples: RNA-seq offers a snapshot of global gene expression (3), techniques such as CLIP-Seq (Crosslinking and Immunoprecipitation Sequencing) provide data on RNA-protein interactions (4), RIL-Seq (RNA Interaction by Ligation and Sequencing) can be used to study RNA-RNA interactions including small RNAs and their targets (5, 6), dRNA-seq and Term-seq map transcriptional start and termination sites (7, 8), and transposon-insertion sequencing (TIS) is used to identify essential genes and study the effects of gene disruption on bacterial fitness in diverse conditions (9). A key feature of these functional genomics technologies is that they provide measurements for every gene in the genome, and so can be reused to answer questions far beyond the initial hypothesis they were generated to address.

An accumulating body of work has demonstrated the utility of integrating functional genomics datasets. This includes a number of studies that have constructed compendia comprising a range of conditions meant to capture natural environments encountered by bacteria, including gene expression and fitness atlases for major human pathogens like *Streptococcus pneumoniae* (10, 11) and *Salmonella* Typhimurium (12, 13). Similar approaches have been used to characterize pathogens across various hosts, such as determining gene requirements for *Legionalla pneumophila* colonization of mammalian and protozoan hosts (14) or differing S. Typhimurium virulence determinants in a range of domesticated animals (15). Yet other studies have combined different technologies. For instance, integration of RNA-seq and TIS data has been used to investigate connections between gene regulation and antibiotic resistance in *Pseudomonas aeruginosa* and *S. pneumoniae* (16, 17), to determine phenotypes for small RNAs and small proteins in *Bacteroides thetaiotaomicron* and *S.* Typhimurium (18, 19), or to identify and characterize a global stress regulator in *Acinetobacter baumannii* (20). All of these studies have produced valuable data that should serve as foundational resources for future work.

However, most of this data remains fragmented across supplementary Excel spreadsheets or as raw sequencing reads on repository servers, preventing easy reuse. Even for researchers with computational experience, finding, (re)processing, and integrating functional genomics data can be a significant challenge. A limited

number of functional genomics studies have included graphical webservers making their data accessible and serving as valuable community resources, notably SalCom for *S*. Typhimuirum (12, 13, 21), Bactome for *P. aeruginosa* (22), PneumoExpress for *S. pneumoniae* (11) and the Theta-Base for *Bacteroides thetaiotaomicron* (19, 23). These servers are often developed to serve data generated for a single study, and generally can't be easily reused or extended for other organisms or types of data. As high-throughput sequencing data continues to accumulate, there is a clear need for infrastructure to support access to and reuse of functional genomics data.

To provide this infrastructure, we introduce Micromix, a cloud-ready platform for sharing and combining functional genomics datasets. Micromix is based on a robust web infrastructure that can support serving hundreds of datasets simultaneously, with an intuitive interface for subsetting and querying the resulting database. Uniquely, Micromix also allows users to upload their own data to enable exploratory analyses in the full context of served data compendia. Through a flexible plug-in system, Micromix can support both new and existing visualization and data interaction tools. As proof-of-concept, we developed an interactive 3D heatmap that can produce publication-quality graphics, and integrated the Clustergrammer (24) biclustering heatmap into Micromix. We present two case studies illustrating the utility of Micromix: first, integrating several *S*. Typhimuirum datasets to examine virulence factor expression and essentiality in a variety of conditions and hosts; and second, interrogating the Theta-Base based on Micromix (19) to investigate regulation of *B. thetaiotaomicron* polysaccharide utilization loci. This first release of Micromix provides a ready solution for serving, integrating, and interacting with data, allowing for the easy construction of community functional genomics resources.

Materials and Methods

Documentation and code availability

The Micromix codebase is freely accessible at: https://github.com/BarquistLab/Micromix. The github repository includes comprehensive guides to installing, using, and modifying Micromix. Additionally, a

tutorial for developing new Micromix plug-ins is available at https://github.com/BarquistLab/pca-plugin.

Site and plugin architecture

The Micromix architecture uses Flask (https://github.com/pallets/flask/) (back-end) and Vue.js (https://vuejs.org/guide/introduction.html) (front-end). Curated datasets are stored on the server as delimited files. Upon dataset selection, a unique session ID is created and the resulting dataset, any transformations and details about any active visualization are stored using MongoDB (https://github.com/mongodb/mongo). Current instances of Micromix have been deployed using Gunicorn (https://readthedocs.org/projects/gunicorn-docs) and Nginx (https://nginx.org/). The Clustergrammer plugin uses the API from the Ma'ayan lab (24), while the HIRI heatmap plugin follows the same front-end and back-end architecture as the main site (Flask, Vue.js) and was developed using WebGL and the Vis.gl framework (https://github.com/visgl).

Functional annotations

Functional annotations related to each bacteria were downloaded using eggNOG-mapper (25). The resulting Gene Ontology terms, KEGG pathways and clusters of orthologous genes (COGs) are extracted using a custom R-script https://github.com/BarquistLab/Micromix, using GO.db (26) and KEGGREST (27) to link pathway identifiers with their descriptions.

Functional genomics data

TPM values from RNA-seq data of different growth and stress conditions for *S.* Typhimurium SL1344 were obtained from supplementary material from (12). The dual RNA-seq time series data was downloaded from (28), and processed using Salmon selective alignment with the dual RNA-seq pipeline (https://nf-co.re/dualrnaseq/1.0.0). TraDIS data was obtained from supplementary material from (15).

Results

Basic functionality of Micromix

Micromix is designed to house microbial functional genomics data, serving as a flexible platform for the development of community resources. In the following sections we describe the basic functionality of Micromix.

A single Micromix instance can serve data for multiple strains or organisms (**Figure 1A**). For each microbe, Micromix can serve curated sets of functional genomics data that are stored on the server as delimited text files that are dynamically loaded into a Mongo database upon user selection. Datasets can be loaded by the user independently (**Figure 1B**), or merged for integrative analyses. Users are also able to upload their own data in a variety of common file formats. Once loaded, datasets can be manipulated using a variety of logical, numeric, and metadata filtering operations described in detail below (**Figure 1C**). Visualization of the manipulated data is provided by integrated plugins that exist as standalone web servers and provide plotting or data exploration services, currently including heatmap and clustering services (**Figure 1D**).

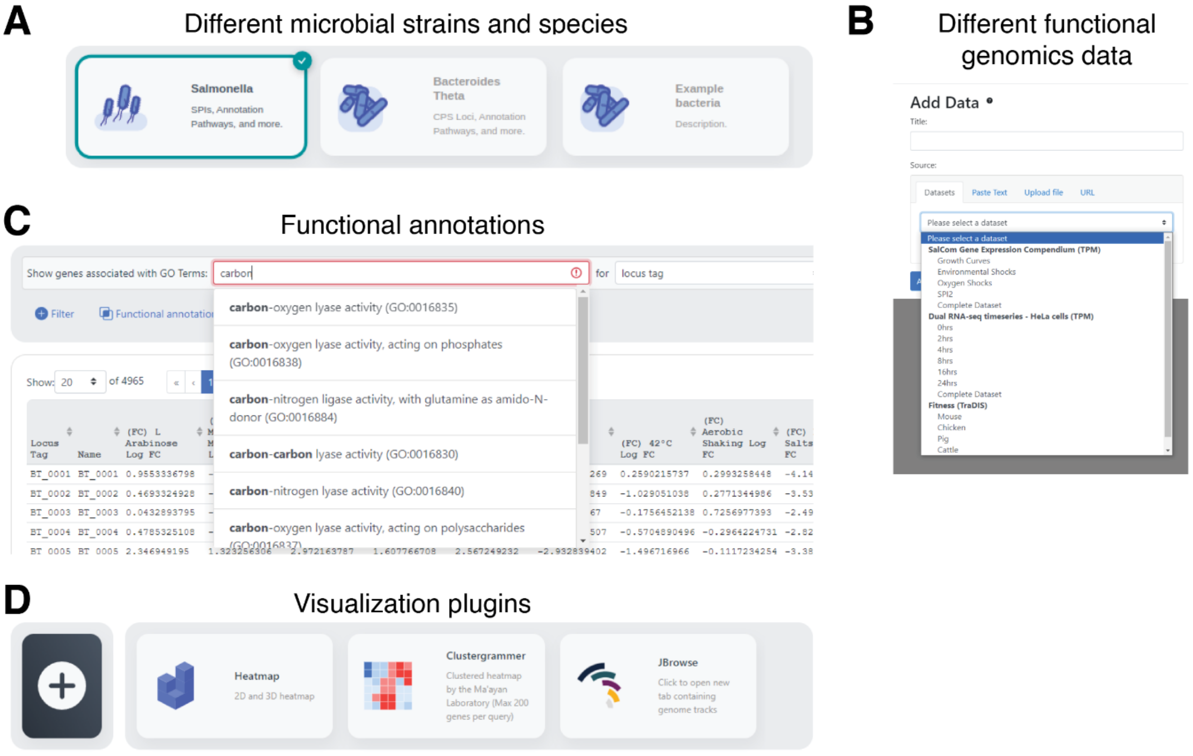

Figure 1: Basic functionality of Micromix. A. Micromix was designed to serve microbial functional genomics data, supporting multiple strains or organisms from a single server instance. **B.** For each organism, multiple datasets can be stored on the server and dynamically loaded and combined by the user. **C**. The user can apply various filters, including pre-loaded functional annotations (e.g. GO terms, KEGG pathways, etc.) **D**. Once the dataset has been loaded and filtered, the resulting dataframe is passed to plugin servers for visualization services.

Merging and interacting with functional genomics data in Micromix

Micromix is built around the concept of a dataframe, a flexible two-dimensional data structure capable of storing any data type (e.g. numeric, character, etc.). Conceptually, a dataframe is similar to the spreadsheets and character separated files that are frequently used to store functional genomics data but can be efficiently manipulated in Python using the pandas data analysis library. Micromix dataframes are organized in gene by condition format, with rows keyed on a unique gene identifier (generally the locus tag defined in the genome annotation) and columns containing measurements from different experiments. Through its graphical user interface (GUI), Micromix provides users with point-and-click access to various data integration, filtering, and manipulation operations.

A single Micromix instance can serve as a repository for an arbitrary number of curated datasets. These might correspond to, for instance, all the data produced by a single study, or a series of related conditions interrogated with the same functional genomics technology. Users can also upload their own data in a variety of common file formats including character-separated value (.csv), tab-delimited text (.txt), and Excel-format files (.xslx).

Micromix provides four basic functionalities for manipulating and sharing dataframes:

1) **Composing dataframes:** multiple datasets can be merged into a single dataframe for exploration and visualization in Micromix. These can include datasets stored on the server and user data, as long as all datasets contain the same gene identifiers. A simple graphical interface allows the user to append new data to either side of the existing dataframe (**Figure 2A**).

2) **Filters and transformations:** Micromix implements three basic types of data filters and transformations: numeric filters, numeric transformations, and annotation filters

(**Figure 2B**). Numeric filters include simple conditional operators like 'less than' or 'not equal to' that can be used to remove dataframe rows. Filters can be applied across individual columns or groups of columns. Numeric transformations provide operations to manipulate the content of loaded dataframes. Available transformations include simple operations such as rounding, conditionally censoring or changing values, and log transforming. Users can also calculate (log) fold-changes within a dataset using a selected column as a reference condition.

Annotation filters depend on the genome of the organism. As a minimal set of annotation filters, we provide an interface to filter rows based on Gene Ontology (GO) (29) term and KEGG (30) pathway annotations. These gene sets result from automatic transfer of annotations using the eggNOG database of orthologous protein groups (31). We provide scripts to parse the results of running eggNOG-mapper (25) on a reference proteome, providing an easy source of annotations during Micromix deployment. Administrators can also provide custom annotations for their organism in a simple JSON format. These might include certain classes of genes of special interest, such as small RNAs, or other genomic features, such as the *Salmonella* pathogenicity islands or *Bacteroides* polysaccharide utilization loci included in our case studies below.

3) **Chaining dataframe manipulations:** The Micromix interface allows multiple filters and transformations to be chained, letting users build up complex queries (**Figure 2C**). For instance, a user could easily build a dataframe for visualization showing log fold-changes or only genes meeting some minimal expression threshold within a pathway or gene set of interest. Filtered and manipulated dataframes can be downloaded by the user in character-separated value or Excel file formats.

4) **Saving and sharing sessions:** Micromix provides users with the ability to save sessions with a simple alphanumeric session ID. The session ID preserves all loaded data, including any user-supplied datasets, filters or transformations that may have been applied, and active visualization plug-ins. Users can also lock their session by clicking on the padlock icon in the toolbar. A locked session can be shared and viewed, but any changes will result in a new session ID being generated. Micromix session IDs allow users to share their data and analyses with lab members, collaborators, or even embedded directly in publications.

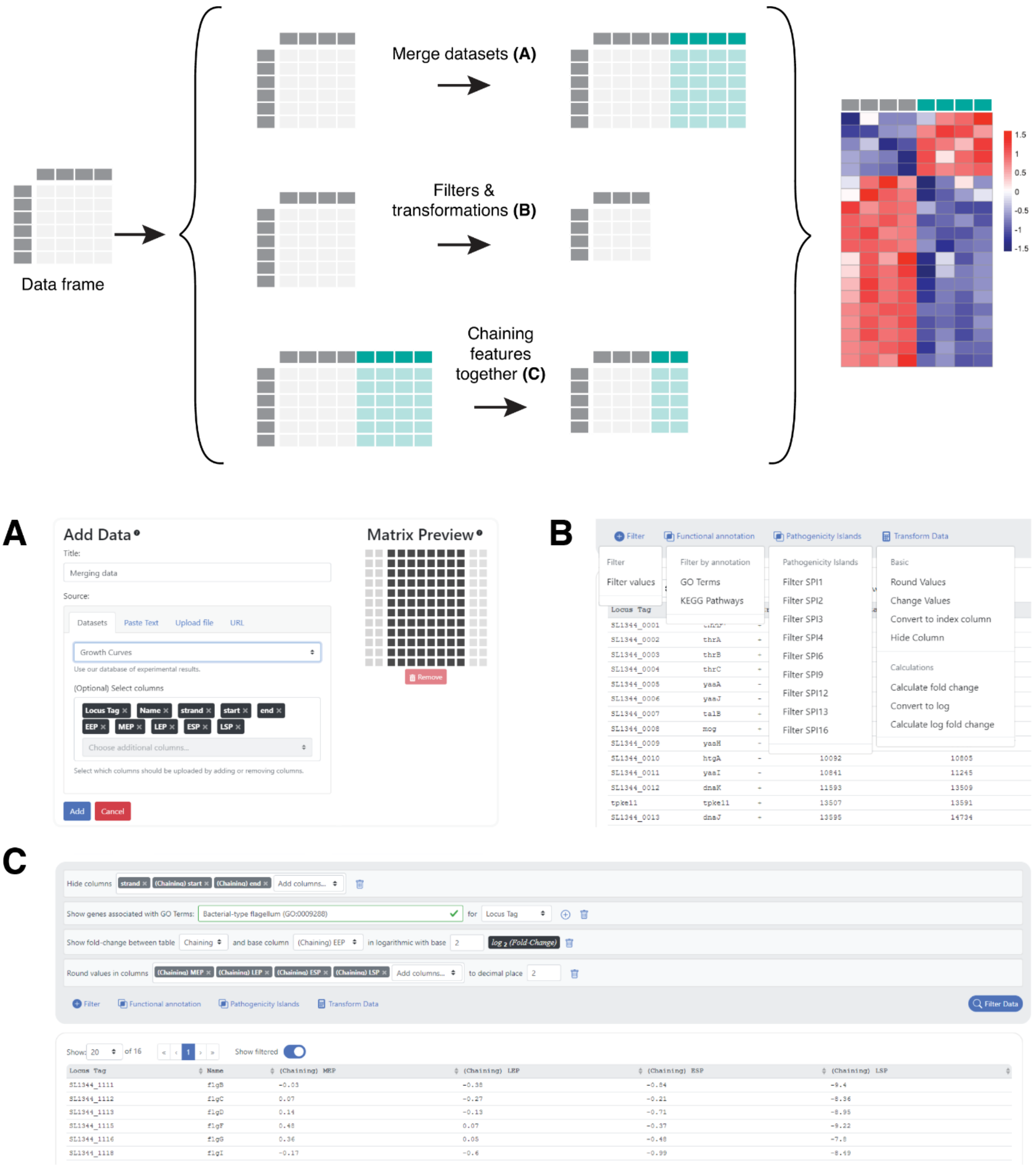

Figure 2: Interacting with dataframes through the Micromix GUI. Micromix allows users to interact with functional genomics data through a variety of dataframe operations. **A.** Users can merge datasets by joining dataframes containing different sets of experimental data. These datasets can be stored server side, or users can upload their own data in a variety of formats. When merging datasets, the matrix preview shows the current data as black squares, and gray squares where additional data can be appended. **B.** Numeric and annotation filters can be used to subset dataframes for gene sets of interest, while transformations can be used to manipulate and scale data. **C.** Filters and transformations can be chained together to produce highly customizable queries.

High performance cloud-ready infrastructure to explore microbial ‘omics data

Micromix was designed to be capable of running on distributed cloud infrastructure, and consists of four major components (**Figure 3**): the Micromix server consisting of a Python backend and Vue.js web interface, a Mongo database (MongoDB) server to store data and session information, and visualization plugins that also run as independent servers. While all components can be run on a single physical web server, the distributed architecture of Micromix allows individual components to be run independently on commercial or academic cloud infrastructure, enabling access for labs that may not have or wish to maintain their own server hardware.

The core of Micromix is a Python backend running Flask, a lightweight web application framework. While Python is a highly abstracted language and hence can suffer from poor performance, Micromix uses the pandas (32) and numpy (33) libraries to efficiently parse, query, merge, and manipulate dataframes housing the underlying data. These libraries are largely implemented in Cython and C, offering high performance even on very large datasets.

The Micromix web interface is built using Vue.js, a lightweight JavaScript framework. Vue.js enables asynchronous communication between the in-browser web interface and backend, providing an interactive application-like experience without page refreshes. The web interface includes dynamic tooltips and help overlays to help users understand site functionality. Visualizations are displayed within an embedded HTML inline frame (iframe), allowing for seamless integration of remote visualization services.

Micromix stores session information and data in a dedicated MongoDB server. Sessions are stored as binary JavaScript Object Notation (JSON) documents, including applied filters, transformations, and any active visualization. These JSON documents can be easily extended, allowing for future development to include additional information, such as organism or dataset metadata. Dataframes are stored within the MongoDB JSON documents in compressed Parquet encoding, reducing both storage requirements and latency between the database and backend.

To benchmark Micromix, we randomly generated numerical matrices with 5,000 rows (representing genes, a typical size for a bacterial genome) and increasing numbers of columns (representing conditions) in steps of 50. Our first release of Micromix can

perform all site functionality using dataframes containing up to 500 conditions, making it suitable to serve large functional genomic datasets.

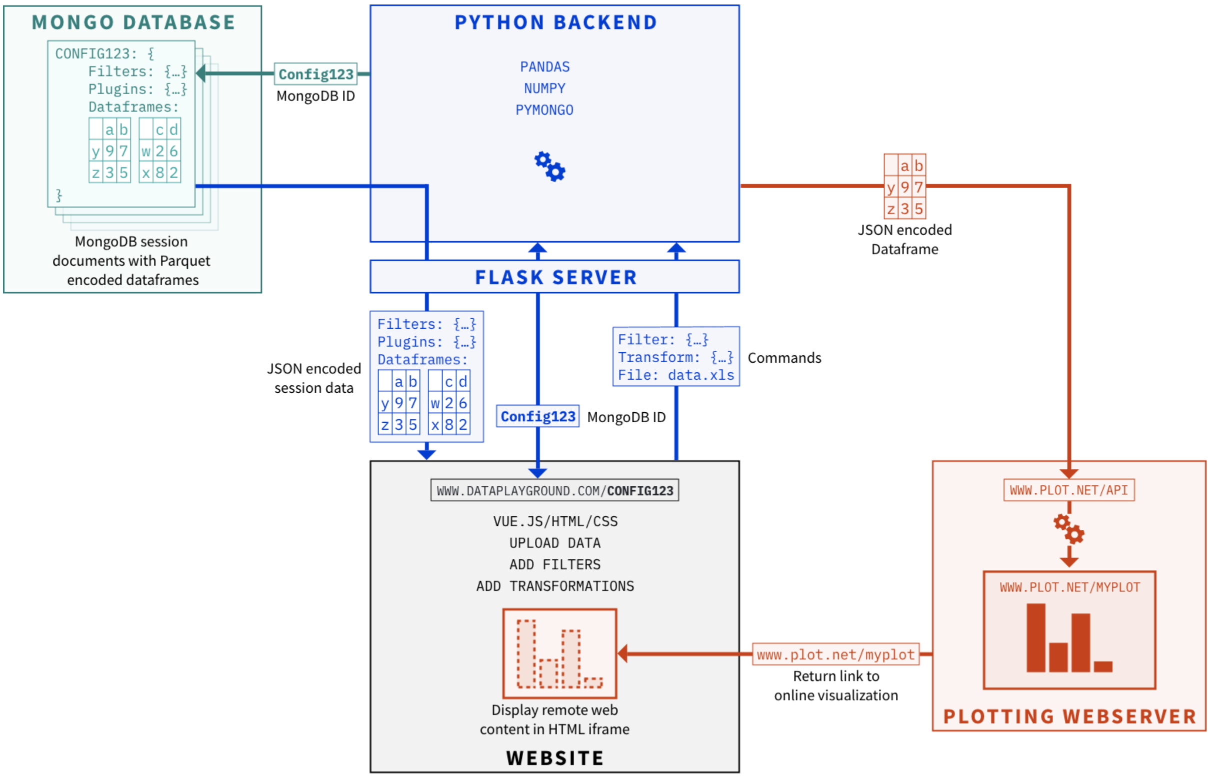

Figure 3: The Micromix architecture. The website (gray) serves as the graphical interface where users can upload and interact with their data. Data and session information are stored as entries in the Mongo database (green) which is accessed through the Python backend (blue). Each session has a unique token number (E.g. “Config123” in the diagram above) that serves as an identifier for a binary JSON document containing user-specified data and filters. When this token ID is appended to the website URL, Micromix will load the corresponding session data from the database. Plotting services (red) are hosted on separate web servers and receive data from the Micromix server through a REST interface. The returned visualization is then embedded in the Micromix interface in an inline frame for display to the user.

A flexible visualization plugin system enables easy extension of Micromix

Micromix relies on independent servers to provide visualization services. This design choice was made for three reasons. First, this allows Micromix to be easily extended without extensive modification of the Micromix code base, as particular visualization services are not an integral part of Micromix. Second, the use of independent servers allows for visualization to be provided as a cloud service, with a single visualization server providing services for potentially many Micromix instances, further distributing

the computational load. Finally, since each visualization server is independent of Micromix, they can easily be reused within other web applications or be run as an independent service, meaning visualization developers are not locked into the Micromix ecosystem and are free to publish and promote their work independently. The only requirements for a Micromix visualization server are that it accepts input data through a REST application programming interface (API) or directly communicates with the MongoDB, and that it returns a webpage appropriate for being embedded in an iframe. REST APIs are compatible with a wide range of web programming languages and frameworks, such as Javascript (with libraries like React), Python (with frameworks like Django), and others. This compatibility allows developers to leverage diverse software independent of Flask and Vue.js to create rich, interactive data visualizations. Plugin interfaces are specified by short Python scripts that define communication between Micromix and the visualization server. We provide a short documented example of plug-in development for a simple PCA server implemented in Plotly.js using direct communication with the MongoDB server at https://github.com/BarquistLab/pca-plugin.

As proof of concept, we have integrated two visualization servers as Micromix plugins. The first plugin is a 3D heatmap application (the HIRI heatmap) we designed as a prototype for exploring large datasets using the Vis.gl framework (https://vis.gl/). The HIRI heatmap uses WebGL, a JavaScript API that provides access to graphics processing unit (GPU) accelerated graphics in a web browser. This graphical acceleration allows users to visualize thousands of heatmap entries in 3D, with real-time rotation and lighting effects. Users can select from a variety of gradient color schemes for their heatmap, manipulate the scale used, and independently color different datasets within the heatmap. A 2D heatmap view can be exported in SVG format suitable for publication (see **Figure 4**) or further editing using popular graphics editors like Adobe Illustrator or Inkscape.

The second plugin provides an interface to Clustergrammer (24), an independent hierarchical clustering server developed and maintained by the Ma'ayan lab. Clustergrammer demonstrates using an existing visualization service where filtered data from Micromix is sent to a REST API, returning an interactive heatmap within Micromix. Clustergrammer provides an interface for interactively exploring clustering results, allowing users to further filter data or reorder rows and columns within the

browser. Due to API restrictions, dataframes are currently limited to 200 rows. The Clustergrammer plugin provides a model for how free standing visualization applications can be easily integrated into Micromix.

Installing and deploying a Micromix server

To allow Micromix to be easily tested and used, we have configured various installation options and provided detailed installation steps to accommodate a wide range of users. For example, users with little to no programming knowledge can download a pre-configured virtual machine and run Micromix locally for testing purposes. Alternatively, Micromix can be installed locally using Docker containers or manually following step by step instructions. We also provide step-by-step instructions for installing and configuring additional server software such as Nginx and Gunicorn necessary to deploy Micromix on a publicly available server or cloud service, allowing the instance to be accessible to a broader community. All necessary instructions, code and download links are accessible at https://github.com/BarquistLab/Micromix.

Case Studies

To demonstrate the utility of this first release of Micromix, we describe two case studies illustrating how Micromix can be used to store and explore bacterial functional genomics data. In the first, we combine gene expression and fitness measurements for the model human and veterinary pathogen *S*. Typhimurium to investigate *Salmonella* pathogenicity island 2 (SPI-2). In the second, we perform cluster analysis of a gene expression atlas for the major human gut commensal *Bacteroides thetaiotaomicron* to identify growth conditions that stimulate the expression of particular polysaccharide expression loci (PULs).

<u>Integrating *Salmonella* functional genomics data across studies with Micromix</u>

Salmonella enterica serovar Typhimurium is a broad host range pathogen, affecting both mammalian and avian hosts, and a major cause of human gastrointestinal illness worldwide (34, 35). Certain lineages of *S*. Typhimurium have been associated with more severe invasive disease epidemics (36), leading to substantial morbidity and mortality (37). *S*. Typhimurium also causes an invasive disease in susceptible mice, which has led to its adoption as a major model organism for investigating host-

pathogen interactions (38). As a result, the lab strain SL1344 (39) and its parent ST4/74 have been extensively studied using functional genomics technologies.

To illustrate the utility of Micromix, we assembled a collection of functional genomics data providing insight into *S*. Typhimurium behavior during infection. In addition to KEGG and GO annotations from eggNOG-mapper, we have included annotations of *Salmonella* pathogenicity islands (SPIs) extracted from the SL1344 genome annotation (40). Pathogenicity islands are horizontally-acquired regions in bacterial genomes that frequently contain genes encoding virulence factors. For functional genomics data, we include a compendium of ST4/74 RNA-seq data in infection-relevant conditions that forms the basis of the SalCom resource (12), SL1344 gene expression from a dual RNA-seq time series taken during infection of HeLa cells (28), and finally fitness measurements for ST4/74 transposon mutants taken in four animal models of infection (15) using transposon-directed insertion-site sequencing (TraDIS).

In this example, we focus on the SPI-2 locus, which encodes a type III secretion system (T3SS) that is crucial for survival and proliferation within host cells (41). Using Micromix, we calculate $\log_2$ fold-changes ($\log_2$FCs) for the RNA-seq data, comparing each set of conditions to a reference condition. For the TraDIS data, the columns containing categorical assignments of fitness effects were used (see Methods). We then filtered for genes within the SPI-2 locus and visualized this data as a heatmap using the HIRI heatmap application (**Figure 4**).

Examination of the heatmap provides an integrated overview of SPI-2 regulation and the fitness effects of gene disruption. Induction of the majority of genes in SPI-2 can be observed in phosphate-carbon-nitrogen (PCN) defined medium (labeled “InSPI2” in the heatmap), designed to emulate key features of the intracellular environment that stimulate SPI-2 expression (42), as well as following bacterial invasion of HeLa cells. We also see a decrease in SPI-2 gene expression over time after the initial induction in HeLa cells, which has been previously described (28). A notable exception to the induction of SPI-2 genes is the *ttr* gene cluster, which encodes genes involved in respiration on tetrathionate (43), a key electron acceptor for *S* Typhimurium in the inflamed mammalian gut (44).

TraDIS coverage of SPI-2 genes is sparse, as an extreme bottleneck during gastrointestinal infection (45) limits the number of mutants that can be screened

simultaneously in the orally inoculated porcine, poultry, and cattle models (15). However, a number of interesting features can still be seen. Insertions in five genes are attenuating across all infection models examined, indicating that gene disruption by transposon insertion results in reduced fitness. These include insertions in *ssrB*, encoding the essential transcriptional activator of SPI-2 gene expression (46), *ssaQ*, encoding a component of the T3SS C-ring essential for secretion (47), *sseC*, encoding an effector translocon protein (48), and *sseG* and *sifA*, encoding SPI-2 effector proteins (49, 50). We also observe a number of effector proteins (*slrP*, *sopD2*, *sseI*, *pipB2*, and *sseK1*) where mutations appear attenuating in gastrointestinal models of infection but not in the tail vein inoculated mouse model, suggesting a primary role in promoting *S*. Typhimurium survival in the gut. PipB2, in particular, is translocated into host cells via both T3SS1 and T3SS2 systems. This dual translocation capability is crucial for intracellular survival, as PipB2 modulates the kinesin-1 motor complex, aiding in the positioning and movement of *Salmonella*-containing vacuoles (SCVs) and thereby enhancing the pathogen's ability to survive and proliferate within host cells (51). Additionally, we observe no phenotype for disruption of two SPI-2 effectors (*sspH2* and *gogB*) despite having fitness measurements in multiple models, possibly indicating functional redundancy in the SPI-2 effector network (52).

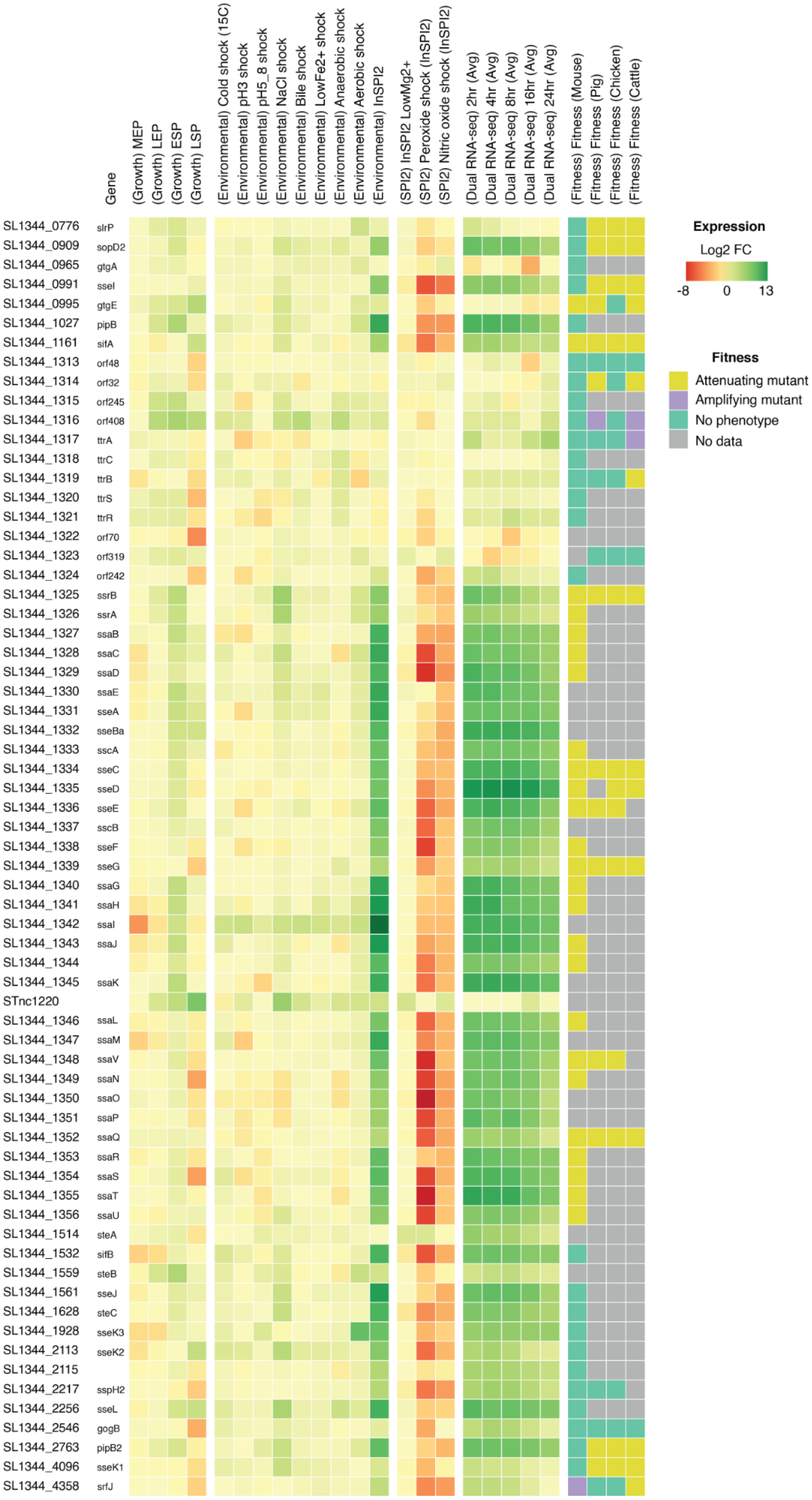
Gene
(Growth) MEP
(Growth) LEP
(Growth) ESP
(Growth) LSP
(Environmental) Cold shock (15C)
(Environmental) pH3 shock
(Environmental) pH5_8 shock
(Environmental) NaCl shock
(Environmental) Bile shock
(Environmental) LowFe2+ shock
(Environmental) Anaerobic shock
(Environmental) Aerobic shock
(Environmental) InSPI2
(SPI2) InSPI2 LowMg2+
(SPI2) Peroxide shock (InSPI2)
(SPI2) Nitric oxide shock (InSPI2)
(Dual RNA-seq) 2hr (Avg)
(Dual RNA-seq) 4hr (Avg)
(Dual RNA-seq) 8hr (Avg)
(Dual RNA-seq) 16hr (Avg)
(Dual RNA-seq) 24hr (Avg)
(Fitness) Fitness (Mouse)
(Fitness) Fitness (Pig)
(Fitness) Fitness (Chicken)
(Fitness) Fitness (Cattle)
SL1344_0776 slrP
SL1344_0909 sopD2
SL1344_0965 gtgA
SL1344_0991 sseI
SL1344_0995 gtgE
SL1344_1027 pipB
SL1344_1161 sifA
SL1344_1313 orf48
SL1344_1314 orf32
SL1344_1315 orf245
SL1344_1316 orf408
SL1344_1317 ttrA
SL1344_1318 ttrC
SL1344_1319 ttrB
SL1344_1320 ttrS
SL1344_1321 ttrR
SL1344_1322 orf70
SL1344_1323 orf319
SL1344_1324 orf242
SL1344_1325 ssrB
SL1344_1326 ssrA
SL1344_1327 ssaB
SL1344_1328 ssaC
SL1344_1329 ssaD
SL1344_1330 ssaE
SL1344_1331 sseA
SL1344_1332 sseBa
SL1344_1333 sscA
SL1344_1334 sseC
SL1344_1335 sseD
SL1344_1336 sseE
SL1344_1337 sscB
SL1344_1338 sseF
SL1344_1339 sseG
SL1344_1340 ssaG
SL1344_1341 ssaH
SL1344_1342 ssaI
SL1344_1343 ssaJ
SL1344_1344
SL1344_1345 ssaK
STnc1220
SL1344_1346 ssaL
SL1344_1347 ssaM
SL1344_1348 ssaV
SL1344_1349 ssaN
SL1344_1350 ssaO
SL1344_1351 ssaP
SL1344_1352 ssaQ
SL1344_1353 ssaR
SL1344_1354 ssaS
SL1344_1355 ssaT
SL1344_1356 ssaU
SL1344_1514 steA
SL1344_1532 sifB
SL1344_1559 steB
SL1344_1561 sseJ
SL1344_1628 steC
SL1344_1928 sseK3
SL1344_2113 sseK2
SL1344_2115
SL1344_2217 sspH2
SL1344_2256 sseL
SL1344_2546 gogB
SL1344_2763 pipB2
SL1344_4096 sseK1
SL1344_4358 srfJ
Expression
Log2 FC
-8
0
13
Fitness
Attenuating mutant
Amplifying mutant
No phenotype
No data

Figure 4: Combining functional genomics datasets to investigate *Salmonella* Pathogenicity Island 2 (SPI-2). Data was retrieved from the supplementary information of RNA-seq (12), dual RNA-seq (28), and TraDIS (15) studies of *Salmonella* in infection-relevant conditions and during infection of a variety of hosts. Log2 fold-changes (log2FCs) were calculated for the RNA-seq and dual RNA-seq datasets, comparing each set of conditions to a reference condition. The four growth phases are relative to early exponential phase (EEP), while environmental shocks are relative to mid exponential phase (MEP) where each of the shocks were performed. SPI2 conditions are relative to InSPI2, and the dual RNA-seq time course is relative to the uninfected cells. TraDIS data was summarized to categorical fitness classifications. The resulting heatmap was directly created using Micromix with the exception of the legend, which was manually added. The locked Micromix session associated with this heatmap can be viewed at: https://micromix.helmholtz-hiri.de/salmonella/?config=667d3789a227c0c978572e3c.

The Theta-Base RNA-seq compendium provides a tool for hypothesis development for the major human commensal *Bacteroides thetaiotaomicron*

As a second example, we illustrate the use of Theta-Base 2.0, an RNA-seq compendium we recently introduced for *B. thetaiotaomicron (19)*. *B. thetaiotaomicron* is a Gram-negative obligate anaerobe and common human gut commensal that has been developed as a model organism for the study of the gut microbiota (53, 54)*. B. thetaiotaomicron* is particularly known for its ability to metabolize a wide range of complex carbohydrates including dietary fibers and host glycans. These metabolic capabilities are mediated by a large collection of polysaccharide utilization loci (PULs) each encoding genes for the detection and metabolism of a particular range of substrates (55). The Theta-Base includes RNA-seq data for 16 different conditions and includes annotations for PULs from PULDB (56) as well as capsular polysaccharide synthesis (CPS) loci, conjugative transposons, and non-coding RNAs.

To demonstrate the utility of the Theta-Base, we first examine a PUL with known substrate and inducing conditions, PUL57. PUL57 is induced by host glycosaminoglycans including chondroitin sulfate and hyaluronic acid, and plays a significant role in their degradation (57). We filtered for these genes using Micromix and visualized the results using Clustergrammer (24) for hierarchical biclustering of genes and conditions (**Figure 5A**). The resulting heatmap showed upregulation of PUL57 in medium supplemented with mucin from porcine stomach in agreement with previous findings (57). We then applied the same procedure to an uncharacterized PUL, PUL29, where the inducing condition is unknown. Here we observe the greatest

expression in medium supplemented with bile salts, indicating that they may serve as an inducing signal for PUL29. The locked Micromix session associated with this heatmap can be viewed at: https://micromix.helmholtz-hiri.de/bacteroides/?config=667e67422e5a30d66f793d96.

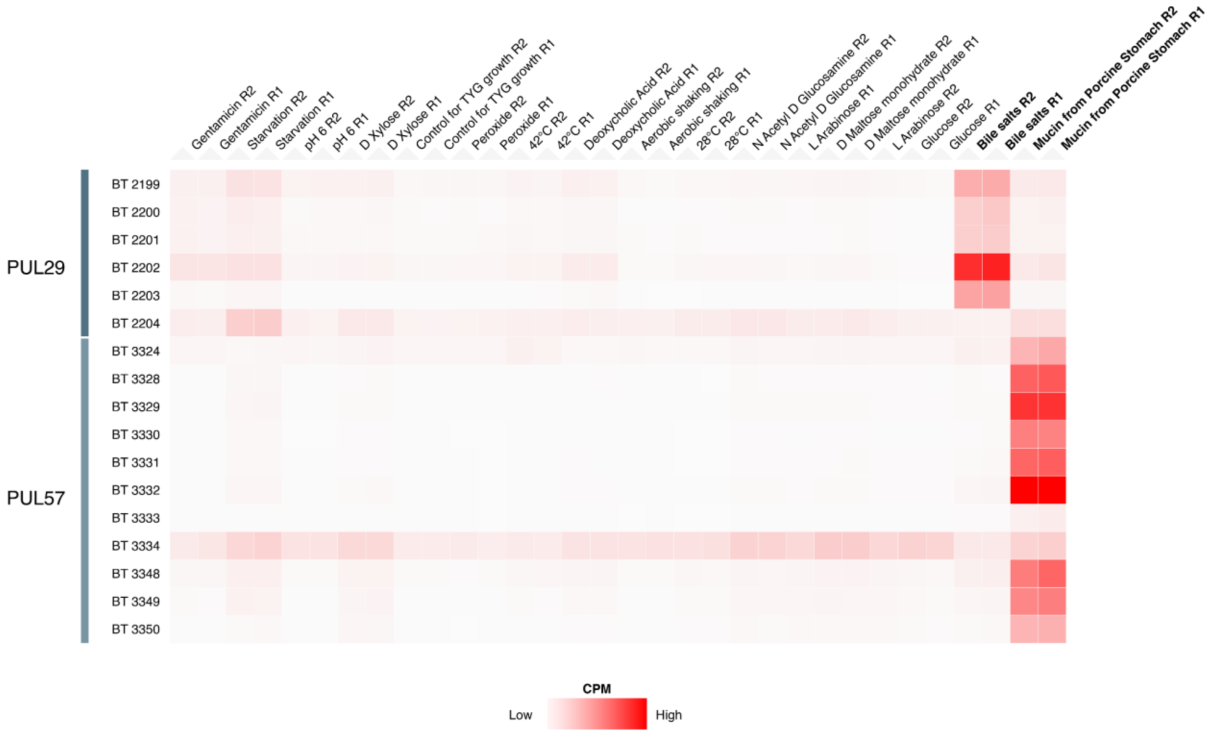

Figure 5: Investigating polysaccharide utilization locus expression in *Bacteroides thetaiotaomicron.* Expression of genes in the PUL29 and PUL57 loci (rows) across conditions (columns) is visualized in counts per million (CPM) and biclustered in Micromix using Clustergrammer (24). PUL29 genes show the highest expression in medium supplemented with bile salts, while PUL57 genes show the highest expression in medium supplemented with mucin from porcine stomach

Discussion

Here we have introduced Micromix, a software platform that enables the construction of web-accessible functional genomics compendia for bacteria. In this first public release, we have provided a foundation for the development of functional genomics community resources. We hope that Micromix will become a nucleating platform for communities working on a variety of microbes to collaboratively build and share comprehensive functional genomic compendia.

We have identified several areas for future development of Micromix. First among these is incorporating and making accessible additional metadata. Currently, experimental metadata is solely provided by an experiment title for each data set. As the size of compendia continue to increase this will become a major limitation; already compendia produced for *Escherichia coli* (58) and *Pseudomonas aeruginoa* (59) exceed 1000 samples. Experimental metadata may also help with the interpretation of visualizations, such as grouping in PCA, or clustering within heatmaps. Our flexible database architecture makes future incorporation of information on e.g. strain, growth condition, or experimental treatment straight-forward, and filters could be added to the interface to make this metadata queryable. However, metadata curation will be a major challenge. While some compendia construction pipelines already automatically collect metadata from e.g. the Gene Expression Omnibus (GEO), this data is often incomplete or incorrect requiring manual curation (59). This may present opportunities to develop new automated or semi-automated curation approaches building on recent advances in natural language processing.

Making gene metadata accessible is also a priority area for development. Our database currently contains associations for genes with COG categories, KEGG pathways (30), and GO terms (29) derived from eggNOG (25), but this information is not accessible to the user except through applying search filters. Making this information accessible through the GUI would allow users to easily explore genes with interesting expression patterns, and could be augmented with links to additional external resources such as InterPro (60) or Rfam (61). Explicit gene metadata could also contain orthology relationships between genomes, making Micromix suitable for application to datasets investigating differences in gene expression and essentiality between related strains. Recent work has shown that even closely related strains can differ significantly in their essential gene complement (62–64), requirements for survival in different conditions (65, 66), and gene expression (67–69), and being able to dynamically switch between reference strains would be a major boon for those working on clinical or environmental isolates.

Finally, this initial release of Micromix has focused on establishing basic usability, and has not undergone systematic optimization. Currently, datasets are limited in size to ~500 columns before Micromix’s performance begins to degrade. While this is more than adequate for typical compendia generated in a single study, it is a limitation when

considering comprehensive resources for well-studied organisms. We believe that this performance can be substantially improved with thorough profiling of Micormix and its constituent components. However, emerging technologies like single cell RNA-seq (70) are likely to soon lead to datasets routinely containing 10's or 100's of thousands of columns. This is likely to lead to both technical challenges in maintaining an interactive interface, as well as the need to consider new ways of reducing the dimensionality of the data to make it understandable to the user without removing important variation.

In summary, Micromix provides a foundation to create functional genomics compendia for bacteria. We have intentionally designed Micromix to be easily deployed and extended, and we look forward to building and supporting a vibrant community of developers and users.

Acknowledgments

We would like to thank Michael Kütt for assistance with deploying and troubleshooting the Micromix and heatmap servers.

Funding

This work was supported by the Bavarian State Ministry for Science and the Arts through the research network bayresq.net and an NSERC Discovery Grant (RGPIN-2024-04305).

Conflict of interest statement. None declared.

Availability of source code and requirements

Project name: Micromix

Project homepage: http://micromix.systems

Operating system: Linux

License: GPL-3.0

Data availability

No new data was produced in the course of this work.

References

1. Hör,J., Gorski,S.A. and Vogel,J. (2018) Bacterial RNA Biology on a Genome Scale. *Mol. Cell*, **70**, 785–799.

2. Perez-Sepulveda,B.M. and Hinton,J.C.D. (2018) Functional Transcriptomics for Bacterial Gene Detectives. *Microbiol Spectr*, **6**.

3. Colgan,A.M., Cameron,A.D. and Kröger,C. (2017) If it transcribes, we can sequence it: mining the complexities of host-pathogen-environment interactions using RNA-seq. *Curr. Opin. Microbiol.*, **36**, 37–46.

4. Saliba,A.-E., C Santos,S. and Vogel,J. (2017) New RNA-seq approaches for the study of bacterial pathogens. *Curr. Opin. Microbiol.*, **35**, 78–87.

5. Melamed,S., Peer,A., Faigenbaum-Romm,R., Gatt,Y.E., Reiss,N., Bar,A., Altuvia,Y., Argaman,L. and Margalit,H. (2016) Global Mapping of Small RNA-Target Interactions in Bacteria. *Mol. Cell*, **63**, 884–897.

6. Melamed,S. (2020) New sequencing methodologies reveal interplay between multiple RNA-binding proteins and their RNAs. *Curr. Genet.*, 10.1007/s00294-020-01066-y.

7. Sharma,C.M., Hoffmann,S., Darfeuille,F., Reignier,J., Findeiss,S., Sittka,A., Chabas,S., Reiche,K., Hackermüller,J., Reinhardt,R., *et al.* (2010) The primary transcriptome of the major human pathogen Helicobacter pylori. *Nature*, **464**, 250–255.

8. Dar,D., Shamir,M., Mellin,J.R., Koutero,M., Stern-Ginossar,N., Cossart,P. and Sorek,R. (2016) Term-seq reveals abundant ribo-regulation of antibiotics resistance in bacteria. *Science*, **352**, aad9822.

9. Cain,A.K., Barquist,L., Goodman,A.L., Paulsen,I.T., Parkhill,J. and van Opijnen,T. (2020) A decade of advances in transposon-insertion sequencing. *Nat. Rev. Genet.*, 10.1038/s41576-020-0244-x.

10. van Opijnen,T. and Camilli,A. (2012) A fine scale phenotype-genotype virulence map of a bacterial pathogen. *Genome Res.*, **22**, 2541–2551.

11. Aprianto,R., Slager,J., Holsappel,S. and Veening,J.-W. (2018) High-resolution analysis of the pneumococcal transcriptome under a wide range of infection-relevant conditions. *Nucleic Acids Res.*, **46**, 9990–10006.

12. Kröger,C., Colgan,A., Srikumar,S., Händler,K., Sivasankaran,S.K., Hammarlöf,D.L., Canals,R., Grissom,J.E., Conway,T., Hokamp,K., *et al.* (2013) An infection-relevant transcriptomic compendium for Salmonella enterica Serovar Typhimurium. *Cell Host*

Microbe, **14**, 683–695.

13. Colgan,A.M., Kröger,C., Diard,M., Hardt,W.-D., Puente,J.L., Sivasankaran,S.K., Hokamp,K. and Hinton,J.C.D. (2016) The Impact of 18 Ancestral and Horizontally-Acquired Regulatory Proteins upon the Transcriptome and sRNA Landscape of Salmonella enterica serovar Typhimurium. *PLoS Genet.*, **12**, e1006258.

14. Shames,S.R., Liu,L., Havey,J.C., Schofield,W.B., Goodman,A.L. and Roy,C.R. (2017) Multiple Legionella pneumophila effector virulence phenotypes revealed through high-throughput analysis of targeted mutant libraries. *Proc. Natl. Acad. Sci. U. S. A.*, **114**, E10446–E10454.

15. Chaudhuri,R.R., Morgan,E., Peters,S.E., Pleasance,S.J., Hudson,D.L., Davies,H.M., Wang,J., van Diemen,P.M., Buckley,A.M., Bowen,A.J., *et al.* (2013) Comprehensive assignment of roles for Salmonella typhimurium genes in intestinal colonization of food-producing animals. *PLoS Genet.*, **9**, e1003456.

16. Murray,J.L., Kwon,T., Marcotte,E.M. and Whiteley,M. (2015) Intrinsic Antimicrobial Resistance Determinants in the Superbug Pseudomonas aeruginosa. *MBio*, **6**, e01603–15.

17. Jensen,P.A., Zhu,Z. and van Opijnen,T. (2017) Antibiotics Disrupt Coordination between Transcriptional and Phenotypic Stress Responses in Pathogenic Bacteria. *Cell Rep.*, **20**, 1705–1716.

18. Venturini,E., Svensson,S.L., Maaß,S., Gelhausen,R., Eggenhofer,F., Li,L., Cain,A.K., Parkhill,J., Becher,D., Backofen,R., *et al.* (2020) A global data-driven census of Salmonella small proteins and their potential functions in bacterial virulence. *microLife*, **1**.

19. Ryan,D., Bornet,E., Prezza,G., Alampalli,S.V., Franco de Carvalho,T., Felchle,H., Ebbecke,T., Hayward,R.J., Deutschbauer,A.M., Barquist,L., *et al.* (2024) An expanded transcriptome atlas for Bacteroides thetaiotaomicron reveals a small RNA that modulates tetracycline sensitivity. *Nat Microbiol*, 10.1038/s41564-024-01642-9.

20. Maharjan,R.P., Sullivan,G.J., Adams,F.G., Shah,B.S., Hawkey,J., Delgado,N., Semenec,L., Dinh,H., Li,L., Short,F.L., *et al.* (2023) DksA is a conserved master regulator of stress response in Acinetobacter baumannii. *Nucleic Acids Res.*, **51**, 6101–6119.

21. Srikumar,S., Kröger,C., Hébrard,M., Colgan,A., Owen,S.V., Sivasankaran,S.K., Cameron,A.D.S., Hokamp,K. and Hinton,J.C.D. (2015) RNA-seq Brings New Insights to the Intra-Macrophage Transcriptome of Salmonella Typhimurium. *PLoS Pathog.*, **11**, e1005262.

22. Dötsch,A., Schniederjans,M., Khaledi,A., Hornischer,K., Schulz,S., Bielecka,A., Eckweiler,D., Pohl,S. and Häussler,S. (2015) The Pseudomonas aeruginosa Transcriptional Landscape Is Shaped by Environmental Heterogeneity and Genetic Variation. *MBio*, **6**, e00749.

23. Ryan,D., Jenniches,L., Reichardt,S., Barquist,L. and Westermann,A.J. (2020) A high-resolution transcriptome map identifies small RNA regulation of metabolism in the gut microbe Bacteroides thetaiotaomicron. *Nat. Commun.*, **11**, 3557.

24. Fernandez,N.F., Gundersen,G.W., Rahman,A., Grimes,M.L., Rikova,K., Hornbeck,P. and Ma'ayan,A. (2017) Clustergrammer, a web-based heatmap visualization and

analysis tool for high-dimensional biological data. *Sci Data*, **4**, 170151.

25. Cantalapiedra,C.P., Hernández-Plaza,A., Letunic,I., Bork,P. and Huerta-Cepas,J. (2021) eggNOG-mapper v2: Functional Annotation, Orthology Assignments, and Domain Prediction at the Metagenomic Scale. *Mol. Biol. Evol.*, **38**, 5825–5829.

26. Carlson,M. (2019) GO.Db: A set of annotation maps describing the entire Gene Ontology assembled using data from GO. *Bioconductor*.

27. Tenenbaum,D. and Maintainer,B. (2022) KEGGREST. *Bioconductor*.

28. Westermann,A.J., Förstner,K.U., Amman,F., Barquist,L., Chao,Y., Schulte,L.N., Müller,L., Reinhardt,R., Stadler,P.F. and Vogel,J. (2016) Dual RNA-seq unveils noncoding RNA functions in host-pathogen interactions. *Nature*, **529**, 496–501.

29. Gene Ontology Consortium (2021) The Gene Ontology resource: enriching a GOld mine. *Nucleic Acids Res.*, **49**, D325–D334.

30. Kanehisa,M., Furumichi,M., Sato,Y., Kawashima,M. and Ishiguro-Watanabe,M. (2023) KEGG for taxonomy-based analysis of pathways and genomes. *Nucleic Acids Res.*, **51**, D587–D592.

31. Hernández-Plaza,A., Szklarczyk,D., Botas,J., Cantalapiedra,C.P., Giner-Lamia,J., Mende,D.R., Kirsch,R., Rattei,T., Letunic,I., Jensen,L.J., *et al.* (2023) eggNOG 6.0: enabling comparative genomics across 12 535 organisms. *Nucleic Acids Res.*, **51**, D389–D394.

32. McKinney,W. (2011) pandas: a foundational Python library for data analysis and statistics. *Python for High Performance and Scientific Computing*, **14**, 1–9.

33. Harris,C.R., Millman,K.J., van der Walt,S.J., Gommers,R., Virtanen,P., Cournapeau,D., Wieser,E., Taylor,J., Berg,S., Smith,N.J., *et al.* (2020) Array programming with NumPy. *Nature*, **585**, 357–362.

34. European Food Safety Authority and European Centre for Disease Prevention and Control (EFSA and ECDC) (2018) The European Union summary report on trends and sources of zoonoses, zoonotic agents and food-borne outbreaks in 2017. *EFSA J.*, **16**, e05500.

35. Centers for Disease Control and Prevention (2018) National enteric disease surveillance: salmonella annual report.

36. Feasey,N.A., Dougan,G., Kingsley,R.A., Heyderman,R.S. and Gordon,M.A. (2012) Invasive non-typhoidal salmonella disease: an emerging and neglected tropical disease in Africa. *Lancet*, **379**, 2489–2499.

37. Stanaway,J.D., Parisi,A., Sarkar,K., Blacker,B.F., Reiner,R.C., Hay,S.I., Nixon,M.R., Dolecek,C., James,S.L., Mokdad,A.H., *et al.* (2019) The global burden of non-typhoidal salmonella invasive disease: a systematic analysis for the Global Burden of Disease Study 2017. *Lancet Infect. Dis.*, **19**, 1312–1324.

38. Tsolis,R.M., Xavier,M.N., Santos,R.L. and Bäumler,A.J. (2011) How to become a top model: impact of animal experimentation on human Salmonella disease research. *Infect. Immun.*, **79**, 1806–1814.

39. Hoiseth,S.K. and Stocker,B.A. (1981) Aromatic-dependent Salmonella typhimurium are non-virulent and effective as live vaccines. *Nature*, **291**, 238–239.

40. Kröger,C., Dillon,S.C., Cameron,A.D.S., Papenfort,K., Sivasankaran,S.K., Hokamp,K., Chao,Y., Sittka,A., Hébrard,M., Händler,K., *et al.* (2012) The transcriptional landscape and small RNAs of *Salmonella enterica* serovar Typhimurium. *Proc. Natl. Acad. Sci. U. S. A.*, **109**, E1277–86.

41. Hensel,M. (2000) Salmonella pathogenicity island 2. *Mol. Microbiol.*, **36**, 1015–1023.

42. Löber,S., Jäckel,D., Kaiser,N. and Hensel,M. (2006) Regulation of Salmonella pathogenicity island 2 genes by independent environmental signals. *Int. J. Med. Microbiol.*, **296**, 435–447.

43. Price-Carter,M., Tingey,J., Bobik,T.A. and Roth,J.R. (2001) The alternative electron acceptor tetrathionate supports B12-dependent anaerobic growth of Salmonella enterica serovar typhimurium on ethanolamine or 1,2-propanediol. *J. Bacteriol.*, **183**, 2463–2475.

44. Winter,S.E., Thiennimitr,P., Winter,M.G., Butler,B.P., Huseby,D.L., Crawford,R.W., Russell,J.M., Bevins,C.L., Adams,L.G., Tsolis,R.M., *et al.* (2010) Gut inflammation provides a respiratory electron acceptor for Salmonella. *Nature*, **467**, 426–429.

45. Maier,L., Diard,M., Sellin,M.E., Chouffane,E.-S., Trautwein-Weidner,K., Periaswamy,B., Slack,E., Dolowschiak,T., Stecher,B., Loverdo,C., *et al.* (2014) Granulocytes impose a tight bottleneck upon the gut luminal pathogen population during Salmonella typhimurium colitis. *PLoS Pathog.*, **10**, e1004557.

46. Fass,E. and Groisman,E.A. (2009) Control of Salmonella pathogenicity island-2 gene expression. *Curr. Opin. Microbiol.*, **12**, 199–204.

47. Yu,X.-J., Liu,M., Matthews,S. and Holden,D.W. (2011) Tandem translation generates a chaperone for the Salmonella type III secretion system protein SsaQ. *J. Biol. Chem.*, **286**, 36098–36107.

48. Nikolaus,T., Deiwick,J., Rappl,C., Freeman,J.A., Schröder,W., Miller,S.I. and Hensel,M. (2001) SseBCD proteins are secreted by the type III secretion system of Salmonella pathogenicity island 2 and function as a translocon. *J. Bacteriol.*, **183**, 6036–6045.

49. Salcedo,S.P. and Holden,D.W. (2003) SseG, a virulence protein that targets *Salmonella* to the Golgi network. *EMBO J.*, **22**, 5003–5014.

50. Beuzón,C.R., Méresse,S., Unsworth,K.E., Ruíz-Albert,J., Garvis,S., Waterman,S.R., Ryder,T.A., Boucrot,E. and Holden,D.W. (2000) Salmonella maintains the integrity of its intracellular vacuole through the action of SifA. *EMBO J.*, **19**, 3235–3249.

51. Baisón-Olmo,F., Cardenal-Muñoz,E. and Ramos-Morales,F. (2012) PipB2 is a substrate of the Salmonella pathogenicity island 1-encoded type III secretion system. *Biochem. Biophys. Res. Commun.*, **423**, 240–246.

52. Sanchez-Garrido,J., Ruano-Gallego,D., Choudhary,J.S. and Frankel,G. (2021) The type III secretion system effector network hypothesis. *Trends Microbiol.*, 10.1016/j.tim.2021.10.007.

53. Wexler,A.G. and Goodman,A.L. (2017) An insider's perspective: Bacteroides as a window into the microbiome. *Nat Microbiol*, **2**, 17026.

54. Porter,N.T., Luis,A.S. and Martens,E.C. (2018) Bacteroides thetaiotaomicron. *Trends Microbiol.*, **26**, 966–967.

55. Grondin,J.M., Tamura,K., Déjean,G., Abbott,D.W. and Brumer,H. (2017) Polysaccharide

Utilization Loci: Fueling Microbial Communities. *J. Bacteriol.*, **199**.

56. Terrapon,N., Lombard,V., Drula,É., Lapébie,P., Al-Masaudi,S., Gilbert,H.J. and Henrissat,B. (2018) PULDB: the expanded database of Polysaccharide Utilization Loci. *Nucleic Acids Res.*, **46**, D677–D683.

57. Martens,E.C., Chiang,H.C. and Gordon,J.I. (2008) Mucosal glycan foraging enhances fitness and transmission of a saccharolytic human gut bacterial symbiont. *Cell Host Microbe*, **4**, 447–457.

58. Lamoureux,C.R., Decker,K.T., Sastry,A.V., Rychel,K., Gao,Y., McConn,J.L., Zielinski,D.C. and Palsson,B.O. (2023) A multi-scale expression and regulation knowledge base for Escherichia coli. *Nucleic Acids Res.*, 10.1093/nar/gkad750.

59. Doing,G., Lee,A.J., Neff,S.L., Reiter,T., Holt,J.D., Stanton,B.A., Greene,C.S. and Hogan,D.A. (2023) Computationally Efficient Assembly of Pseudomonas aeruginosa Gene Expression Compendia. *mSystems*, **8**, e0034122.

60. Paysan-Lafosse,T., Blum,M., Chuguransky,S., Grego,T., Pinto,B.L., Salazar,G.A., Bileschi,M.L., Bork,P., Bridge,A., Colwell,L., *et al.* (2022) InterPro in 2022. *Nucleic Acids Res.*, **51**, D418–D427.

61. Kalvari,I., Nawrocki,E.P., Ontiveros-Palacios,N., Argasinska,J., Lamkiewicz,K., Marz,M., Griffiths-Jones,S., Toffano-Nioche,C., Gautheret,D., Weinberg,Z., *et al.* (2020) Rfam 14: expanded coverage of metagenomic, viral and microRNA families. *Nucleic Acids Res.*, **49**, D192–D200.

62. Rousset,F., Cabezas-Caballero,J., Piastra-Facon,F., Fernández-Rodríguez,J., Clermont,O., Denamur,E., Rocha,E.P.C. and Bikard,D. (2021) The impact of genetic diversity on gene essentiality within the Escherichia coli species. *Nat Microbiol*, **6**, 301–312.

63. Rosconi,F., Rudmann,E., Li,J., Surujon,D., Anthony,J., Frank,M., Jones,D.S., Rock,C., Rosch,J.W., Johnston,C.D., *et al.* (2022) A bacterial pan-genome makes gene essentiality strain-dependent and evolvable. *Nat Microbiol*, 10.1038/s41564-022-01208-7.

64. Ghomi,F.A., Langridge,G.C., Cain,A.K., Boinett,C., El Ghany,M.A., Pickard,D.J., Kingsley,R.A., Thomson,N.R., Parkhill,J., Gardner,P.P., *et al.* (2022) High-throughput transposon mutagenesis in the family Enterobacteriaceae reveals core essential genes and rapid turnover of essentiality. *bioRxiv*, 10.1101/2022.10.20.512852.

65. Poulsen,B.E., Yang,R., Clatworthy,A.E., White,T., Osmulski,S.J., Li,L., Penaranda,C., Lander,E.S., Shoresh,N. and Hung,D.T. (2019) Defining the core essential genome of Pseudomonas aeruginosa. *Proc. Natl. Acad. Sci. U. S. A.*, **116**, 10072–10080.

66. Wang,B.X., Leshchiner,D., Luo,L., Tuncel,M., Hokamp,K., Hinton,J.C.D. and Monack,D.M. (2024) High-throughput fitness experiments reveal specific vulnerabilities of human-adapted Salmonella during stress and infection. *Nat. Genet.*, **56**, 1288–1299.

67. Canals,R., Hammarlöf,D.L., Kröger,C., Owen,S.V., Fong,W.Y., Lacharme-Lora,L., Zhu,X., Wenner,N., Carden,S.E., Honeycutt,J., *et al.* (2019) Adding function to the genome of African Salmonella Typhimurium ST313 strain D23580. *PLoS Biol.*, **17**, e3000059.

68. Mika-Gospodorz,B., Giengkam,S., Westermann,A.J., Wongsantichon,J., Kion-

Crosby,W., Chuenklin,S., Wang,L.C., Sunyakumthorn,P., Sobota,R.M., Subbian,S., *et al.* (2020) Dual RNA-seq of Orientia tsutsugamushi informs on host-pathogen interactions for this neglected intracellular human pathogen. *Nat. Commun.*, **11**, 3363.

69. O'Boyle,N., Douce,G.R., Farrell,G., Rattray,N.J.W., Schembri,M.A., Roe,A.J. and Connolly,J.P.R. (2023) Distinct ecological fitness factors coordinated by a conserved Escherichia coli regulator during systemic bloodstream infection. *Proc. Natl. Acad. Sci. U. S. A.*, **120**, e2212175120.

70. Homberger,C., Barquist,L. and Vogel,J. (2022) Ushering in a new era of single-cell transcriptomics in bacteria. *Microlife*, **3**, uqac020.
