# Supplementary material for: Micromix: web infrastructure for visualizing and remixing microbial ‘omics data
Source: Gigascience. 2025 Feb 3;14:giae120. doi: 10.1093/gigascience/giae120 (PMC11788673; doi:10.1093/gigascience/giae120)
Supplement: giae120_Response_to_Reviewer_Comments_Original_Submission [file giae120_response_to_reviewer_comments_original_submission.pdf]

Author's Response To Reviewer Comments

We would like to thank both reviewers for this insightful comments. We have now responded to all comments, including making several modifications to the Micromix server. We have uploaded a revised manuscript with all changes highlight in red. We hope that you now find our manuscript acceptable for publication.

Reviewer #1: This manuscript provides a comprehensive overview of microbial data integration platforms, highlighting key features such as data repositories, visualization tools, and integrative analysis methods, while also showcasing practical applications of Micromix. It offers valuable insights for researchers and developers looking to enhance their understanding of microbial 'omics data management and facilitate the construction of community functional genomics resources. Additionally, the website design is commendable, and the images on GitHub offer clear explanations that help users navigate the platform effectively. It is evident that the authors have invested significant effort into making the software user-friendly. However, there are a few suggestions to further refine and strengthen this work.

We would like to thank the reviewer for their summary and positive comments on our manuscript and Micromix.

Major revisions:

1. Please provide usage statistics for the platform in the last paragraph of the Introduction to demonstrate its popular and impact within the field. On GitHub, the software currently shows 0 stars, 0 forks, and 1 watcher. Due to the non-unique name "Micromix," searches on Google Scholar do not accurately reflect the platform's widespread usage.

While this manuscript serves as the first public release of Micromix, and so we do not currently have a large user base, we have reported statistics for the Theta-Base released earlier this year based on Micromix. This has served over 11000 unique visitors, excluding crawlers.
----

2. While using the examples provided on the website, I noticed that when the data volume exceeds 100 entries, the performance of the heatmap and cluster grammer becomes noticeably slow, which detracts from the overall user experience. This is a common issue with web applications, but I recommend including an estimated time for results display or a progress bar to set clear expectations for task completion.

Thank you for this comment, we have added loading notifications for all tasks that may take some time now.
----

3. Please add brief annotations for the Pathogenicity Islands on your website.

The SPI nomenclature currently used on the website is generally used in the Salmonella community. Often these islands contain genes serving either multiple functions or unknown functions, so further annotation would currently be difficult or potentially misleading. We hope to provide further access to metadata in future releases.
----

4. The website allows users to upload their own data. I recommend providing sample data in the upload or paste section and indicating which columns are mandatory, to help users prepare their data more

effectively.

We now provide example data in the github in this section:
https://github.com/BarquistLab/Micromix/blob/main/using_micromix.md#uploading-datasets
----

5. I In the numeric filters on your website, only operators like 'less than,' 'not equal to,' and 'more than' can be selected. Please consider adding an option to filter values within a specific range, such as greater than 10 and less than 30.

For technical reasons, this would currently be difficult to implement. We will add it to our list of improvement for future releases.
----

6. Some webserver, such as ImageGP, Wekemo Bioincloud should be compare and discussed to learn some advantage from them.

We have added a brief reference to these servers on line 492.

Minor revisions:

1. The downloaded SVG or PNG heatmaps and clustergrammer images from the website lack a scale legend.

We have added scale legends to our heatmap plug-in. Unfortunately, since clustergrammer is provided by an external service, we can not easily modify the visualization to include a scale legend.
----

2. The clustering rules for cluster grammer images are not clearly defined on the website. Please specify the principles or criteria used for clustering.

These are described in the clustergrammer documentation (https://clustergrammer.readthedocs.io/getting_started.html), linked from the ? icon in the top left corner of the embedded visualization.
----

3. Please review the manuscript for consistency in species names. For example, in line 58, "S. Typhimurium" should have the genus name italicized. In line 368, it should be "S. Typhimurium," not "S Typhimurium." Additionally, in the Materials and Methods section, you refer to "S. Typhimurium SL1344," while other sections refer to "S. Typhimurium." Note that "S. Typhimurium SL1344" is a specific strain of S. Typhimurium, so please ensure consistency throughout the manuscript.

We have corrected the strains included in the materials and methods. All references to individual strains in the main text were correct.
----

4. In line 122, Please provide the full name of TPM.

We have now defined the acronym.

Reviewer #2: The FAIR (finable, accessible, interoperable, and reusable) principle is crucial in open science field. This manuscript focuses on addressing the critical issue of data reuse by introducing Micromix, a web platform designed for the sharing and analysis of microbial omics data. Micromix not only provides an online platform for data visualization, but also empowers users to upload their data for integrative analysis. Users can analyse the gene expression without the coding background. One notable aspect of Micromix is the implementation of a modular visualization system, where visualization services are designed as plugin components rather than being tightly integrated within Micromix itself. This design choice enables users to effortlessly expand the platform with other visualization systems, enhancing flexibility and customization options. Furthermore, the source of Micromix is open access, and authors provide three ways to install and deploy the Micromix. In
conclusion, Micromix not only promotes data reusability and encourages collaboration but also highlights its commitment to advancing open science practices in the field of microbial omics research.

We thank the review for their encouraging comments.

While this manuscript was interesting to read, I have the following concerns:

1 The authors pointed out that "the absence of solutions... supplementary spreadsheets or raw read data left dormant in public repositories..." However, it seems that Micromix primarily concentrates on the expression matrix (like Excel). I am uncertain how Micromix addresses the issue of "raw read data." Can Micromix handle raw sequences for analysis, similar to MG-RAST?

Micromix does not directly solve the problem of processing raw read data. We have deleted the clause in question to avoid confusion.
----

2 As Micromix endeavors to improve the reusability of microbiological data, it raises the question of whether Micromix could potentially collaborate with public data repositories. For instance, could Micromix integrate with these repositories to directly access their data using APIs? Although I know it is hard.

This is a good suggestion, but indeed difficult to the point of almost becoming an entirely new project. We leave this for future work for the moment.
----

3 While the authors offer Virtual Machine, Docker Containers, and Manual installation methods for deploying Micromix, I recommend that they consider providing the Singularity method as well. Many users may lack SUDO access on their service, making Singularity a valuable alternative for deployment.

Due to time limitations, we have not been able to create an additional Singularity container. We believe offering 3 installation options covers most common use cases.
----

4 When I click on S. Typhimurium, a banner appeared stating "Update v1.2.0: Updated query logic". However, the information displayed for Theta-Base v2 indicates "Update v1.1.0: Updated query logic." Does this suggest that they are using different versions of Micromix?

These version numbers tracked internal database changes, and not the software version. We have now removed the banners to avoid confusion.

----

5 I recommend adding a loading animation when users upload a dataset or apply filter conditions. It might be due to my network connection, as there was a delay in the webpage refreshing after I uploaded the data, which made me think that there was a bug.

In response this and the 1st reviewer's similar comment, we have now added loading screens during time-intensive operations.
----

6 While Micromix enables users to upload their data for analysis, I recommend providing an example of the upload file requirements. For instance, clarification on whether the file should include headers and if there are any size limitations for the upload file would be beneficial for users.

We now provide example data in the github in this section:
https://github.com/BarquistLab/Micromix/blob/main/using_micromix.md#uploading-datasets
----

7 It is quite beneficial that Micromix offers the "saving and sharing" feature, particularly the "lock" function. By enabling users to easily share page data without requiring a login, Micromix eliminates the need for developers to create a user system. This simplifies the user experience, allowing for more straightforward and efficient utilization of Micromix.
However, I am curious about how long these user analysis data will be retained. Will there be backup measures in place to ensure data preservation and security?

Since our current servers are largely proof-of-concept, we do not have any explicit policy about retaining data, and leave this as a decision to system administrators. We do include some scripts that can be used for database maintenance should the size of the database become an issue. These could be set up as cronjobs to regularly clear sessions over a certain age, and to preserve locked sessions.
----

8 On GitHub, within the Infrastructure section of the README.md file, the example link (http://micromix.helmholtz-hiri.de/bacteroides/?config=652d4077f7670759f17ae4ba) appears to be malfunctioning, displaying an Unexpected Error: Error: Request failed with status code 500.

Thank you for pointing this out, we have updated the link.
