# Supplementary material for: Micromix: web infrastructure for visualizing and remixing microbial ‘omics data
Source: Gigascience. 2025 Feb 3;14:giae120. doi: 10.1093/gigascience/giae120 (PMC11788673; doi:10.1093/gigascience/giae120)
Supplement: giae120_Reviewer_1_Report_Original_Submission — Yongxin Liu -- 9/2/2024 Reviewed [file giae120_reviewer_1_report_original_submission.pdf]

Reviewer Report

Title: Micromix: web infrastructure for visualizing and remixing microbial 'omics data

Version: Original Submission **Date:** 9/2/2024

Reviewer name: Yongxin Liu

Reviewer Comments to Author:

This manuscript provides a comprehensive overview of microbial data integration platforms, highlighting key features such as data repositories, visualization tools, and integrative analysis methods, while also showcasing practical applications of Micromix. It offers valuable insights for researchers and developers looking to enhance their understanding of microbial 'omics data management and facilitate the construction of community functional genomics resources. Additionally, the website design is commendable, and the images on GitHub offer clear explanations that help users navigate the platform effectively. It is evident that the authors have invested significant effort into making the software user-friendly. However, there are a few suggestions to further refine and strengthen this work.
Major revisions:
1. Please provide usage statistics for the platform in the last paragraph of the Introduction to demonstrate its popular and impact within the field. On GitHub, the software currently shows 0 stars, 0 forks, and 1 watcher. Due to the non-unique name "Micromix," searches on Google Scholar do not accurately reflect the platform's widespread usage.
2. While using the examples provided on the website, I noticed that when the data volume exceeds 100 entries, the performance of the heatmap and cluster grammer becomes noticeably slow, which detracts from the overall user experience. This is a common issue with web applications, but I recommend including an estimated time for results display or a progress bar to set clear expectations for task completion.
3. Please add brief annotations for the Pathogenicity Islands on your website.
4. The website allows users to upload their own data. I recommend providing sample data in the upload or paste section and indicating which columns are mandatory, to help users prepare their data more effectively.
5. I In the numeric filters on your website, only operators like 'less than,' 'not equal to,' and 'more than' can be selected. Please consider adding an option to filter values within a specific range, such as greater than 10 and less than 30.
6. Some webserver, such as ImageGP, Wekemo Bioincloud should be compare and discussed to learn some advantage from them.
Minor revisions:
1. The downloaded SVG or PNG heatmaps and clustergrammer images from the website lack a scale legend.
2. The clustering rules for cluster grammer images are not clearly defined on the website. Please specify the principles or criteria used for clustering.
3. Please review the manuscript for consistency in species names. For example, in line 58, "S.

Typhimurium" should have the genus name italicized. In line 368, it should be "S. Typhimurium," not "S Typhimurium." Additionally, in the Materials and Methods section, you refer to "S. Typhimurium SL1344," while other sections refer to "S. Typhimurium." Note that "S. Typhimurium SL1344" is a specific strain of S. Typhimurium, so please ensure consistency throughout the manuscript.
4. In line 122, Please provide the full name of TPM.

Methods

Are the methods appropriate to the aims of the study, are they well described, and are necessary controls included? Choose an item.

Conclusions

Are the conclusions adequately supported by the data shown? Choose an item.

Reporting Standards

Does the manuscript adhere to the journal's guidelines on minimum standards of reporting? Choose an item.

Choose an item.

Statistics

Are you able to assess all statistics in the manuscript, including the appropriateness of statistical tests used? Choose an item.

Quality of Written English

Please indicate the quality of language in the manuscript: Choose an item.

Declaration of Competing Interests

Please complete a declaration of competing interests, considering the following questions:

- Have you in the past five years received reimbursements, fees, funding, or salary from an organisation that may in any way gain or lose financially from the publication of this manuscript, either now or in the future?
- Do you hold any stocks or shares in an organisation that may in any way gain or lose financially from the publication of this manuscript, either now or in the future?
- Do you hold or are you currently applying for any patents relating to the content of the manuscript?
- Have you received reimbursements, fees, funding, or salary from an organization that holds or has applied for patents relating to the content of the manuscript?

- Do you have any other financial competing interests?
- Do you have any non-financial competing interests in relation to this paper?

If you can answer no to all of the above, write 'I declare that I have no competing interests' below. If your reply is yes to any, please give details below.

'I declare that I have no competing interests

I agree to the open peer review policy of the journal. I understand that my name will be included on my report to the authors and, if the manuscript is accepted for publication, my named report including any attachments I upload will be posted on the website along with the authors' responses. I agree for my report to be made available under an Open Access Creative Commons CC-BY license (http://creativecommons.org/licenses/by/4.0/). I understand that any comments which I do not wish to be included in my named report can be included as confidential comments to the editors, which will not be published.

Choose an item.

To further support our reviewers, we have joined with Publons, where you can gain additional credit to further highlight your hard work (see: https://publons.com/journal/530/gigascience). On publication of this paper, your review will be automatically added to Publons, you can then choose whether or not to claim your Publons credit. I understand this statement.

Yes Choose an item.
