# Supplementary material for: Micromix: web infrastructure for visualizing and remixing microbial ‘omics data
Source: Gigascience. 2025 Feb 3;14:giae120. doi: 10.1093/gigascience/giae120 (PMC11788673; doi:10.1093/gigascience/giae120)
Supplement: giae120_Reviewer_2_Report_Original_Submission — Weiwen Wang -- 10/7/2024 Reviewed [file giae120_reviewer_2_report_original_submission.pdf]

Reviewer Report

Title: Micromix: web infrastructure for visualizing and remixing microbial 'omics data

Version: Original Submission **Date:** 10/7/2024

Reviewer name: Weiwen Wang

Reviewer Comments to Author:

The FAIR (finable, accessible, interoperable, and reusable) principle is crucial in open science field. This manuscript focuses on addressing the critical issue of data reuse by introducing Micromix, a web platform designed for the sharing and analysis of microbial omics data. Micromix not only provides an online platform for data visualization, but also empowers users to upload their data for integrative analysis. Users can analyse the gene expression without the coding background. One notable aspect of Micromix is the implementation of a modular visualization system, where visualization services are designed as plugin components rather than being tightly integrated within Micromix itself. This design choice enables users to effortlessly expand the platform with other visualization systems, enhancing flexibility and customization options. Furthermore, the source of Micromix is open access, and authors provide three ways to install and deploy the Micromix. In conclusion, Micromix not only promotes data reusability and encourages collaboration but also highlights its commitment to advancing open science practices in the field of microbial omics research.

While this manuscript was interesting to read, I have the following concerns:

1 The authors pointed out that "the absence of solutions... supplementary spreadsheets or raw read data left dormant in public repositories..." However, it seems that Micromix primarily concentrates on the expression matrix (like Excel). I am uncertain how Micromix addresses the issue of "raw read data." Can Micromix handle raw sequences for analysis, similar to MG-RAST?

2 As Micromix endeavors to improve the reusability of microbiological data, it raises the question of whether Micromix could potentially collaborate with public data repositories. For instance, could Micromix integrate with these repositories to directly access their data using APIs? Although I know it is hard.

3 While the authors offer Virtual Machine, Docker Containers, and Manual installation methods for deploying Micromix, I recommend that they consider providing the Singularity method as well. Many users may lack SUDO access on their service, making Singularity a valuable alternative for deployment.

4 When I click on S. Typhimurium, a banner appeared stating "Update v1.2.0: Updated query logic". However, the information displayed for Theta-Base v2 indicates "Update v1.1.0: Updated query logic." Does this suggest that they are using different versions of Micromix?

5 I recommend adding a loading animation when users upload a dataset or apply filter conditions. It might be due to my network connection, as there was a delay in the webpage refreshing after I uploaded the data, which made me think that there was a bug.

6 While Micromix enables users to upload their data for analysis, I recommend providing an example of the upload file requirements. For instance, clarification on whether the file should include headers and if there are any size limitations for the upload file would be beneficial for users.

7 It is quite beneficial that Micromix offers the "saving and sharing" feature, particularly the "lock" function. By enabling users to easily share page data without requiring a login, Micromix eliminates the need for developers to create a user system. This simplifies the user experience, allowing for more straightforward and efficient utilization of Micromix.
However, I am curious about how long these user analysis data will be retained. Will there be backup measures in place to ensure data preservation and security?
8 On GitHub, within the Infrastructure section of the README.md file, the example link (http://micromix.helmholtz-hiri.de/bacteroides/?config=652d4077f7670759f17ae4ba) appears to be malfunctioning, displaying an Unexpected Error: Error: Request failed with status code 500.

Methods

Are the methods appropriate to the aims of the study, are they well described, and are necessary controls included? Choose an item.

Conclusions

Are the conclusions adequately supported by the data shown? Choose an item.

Reporting Standards

Does the manuscript adhere to the journal's guidelines on [minimum standards of reporting?] Choose an item.

Choose an item.

Statistics

Are you able to assess all statistics in the manuscript, including the appropriateness of statistical tests used? Choose an item.

Quality of Written English

Please indicate the quality of language in the manuscript: Choose an item.

Declaration of Competing Interests

Please complete a declaration of competing interests, considering the following questions:

- Have you in the past five years received reimbursements, fees, funding, or salary from an organisation that may in any way gain or lose financially from the publication of this manuscript, either now or in the future?
- Do you hold any stocks or shares in an organisation that may in any way gain or lose financially from the publication of this manuscript, either now or in the future?

- Do you hold or are you currently applying for any patents relating to the content of the manuscript?
- Have you received reimbursements, fees, funding, or salary from an organization that holds or has applied for patents relating to the content of the manuscript?
- Do you have any other financial competing interests?
- Do you have any non-financial competing interests in relation to this paper?

If you can answer no to all of the above, write 'I declare that I have no competing interests' below. If your reply is yes to any, please give details below.

I declare that I have no competing interests

I agree to the open peer review policy of the journal. I understand that my name will be included on my report to the authors and, if the manuscript is accepted for publication, my named report including any attachments I upload will be posted on the website along with the authors' responses. I agree for my report to be made available under an Open Access Creative Commons CC-BY license (http://creativecommons.org/licenses/by/4.0/). I understand that any comments which I do not wish to be included in my named report can be included as confidential comments to the editors, which will not be published.

Choose an item.

To further support our reviewers, we have joined with Publons, where you can gain additional credit to further highlight your hard work (see: https://publons.com/journal/530/gigascience). On publication of this paper, your review will be automatically added to Publons, you can then choose whether or not to claim your Publons credit. I understand this statement.

Yes Choose an item.
